# Genetically Engineered Phage Induced Selective H9c2 Cardiomyocytes Patterning in PDMS Microgrooves

**DOI:** 10.3390/ma10080973

**Published:** 2017-08-21

**Authors:** Youngjun Kim, Chunga Kwon, Hojeong Jeon

**Affiliations:** 1 Korea Institute of Science and Technology Europe (KIST-Europe) Forschungsgesellschaft mbH, Campus E 7 1, 66123 Saarbrücken, Germany; c.kwon@kist-europe.de; 2 Center for Biomaterials, Biomedical Research Institute, Korea Institute of Science and Technology (KIST), Hwarangno 14-gil 5, Seongbuk-gu, Seoul 136-791, Korea; jeonhj@kist.re.kr; 3 Department of Biomedical Engineering, University of Science and Technology (UST), Daejeon 34113, Korea

**Keywords:** polydimethylsiloxane (PDMS), micro-patterns, RGD-phage, cell-surface interaction, rat H9c2 cardiomyocytes

## Abstract

A micro-patterned cell adhesive surface was prepared for future design of medical devices. One-dimensional polydimethylsiloxane (PDMS) micro-patterns were prepared by a photolithography process. Afterwards, recombinant filamentous phages that displayed a short binding motif with a cell adhesive peptide (-RGD-) on p8 proteins were immobilized on PDMS microgrooves through simple contact printing to study the cellular response of rat H9c2 cardiomyocyte. While the cell density decreased on PDMS micro-patterns, we observed enhanced cell proliferation and cell to surface interaction on the RGD-phage coated PDMS microgrooves. The RGD-phage coating also supported a better alignment of cell spreading rather than isotropic cell growths as we observed on non-pattered PDMS surface.

## 1. Introduction

For engineering the cell-material interface for controlling cell adhesion, much attention has been given to the development of various micro-patterned environments in which anisotropic design of a scaffold for biomaterials and biomedical applications are used [1,2,3]. Topographical properties of micro-patterning are especially used in cell adhesion, cell shape and cell guidance in organized patterns to regulate proliferation [4], differentiation [5] and morphogenesis [6] of cells. Although micro-patterning is powerful for manipulating extracellular signaling molecules to investigate cell responses, individually separated microgrooves and hydrophobic PDMS cannot provide contact interfaces between cells, which are important for cell-cell and cell-substrate interactions [2,7]. Additionally, cells on the microgroove substrate exhibited significantly lower cell proliferation compared to those on the plane surface [8,9]. Hence, in addition to the surface topography, the improvement of cellular response through chemical surface modification by bio-passive and/or -active coatings has been extensively studied [10]. Such biochemical surface modification may especially help impede early immune responses, including inflammation and encapsulation [11]. For instance, the surface modifications made by fibrin and laminin support the reduction of any possible foreign body responses. Fibrin and laminin are extra cellular matrix proteins (ECMs) and it is known that they are involved in early immune response processes such as inflammation [12]. In addition to early immune responses for ECM, the arginine-glycine-aspartate (RGD) peptide which is recognized by integrin to promote cell adhesion has been investigated to control the cellular response on spreading, migration, morphology and orientation [13,14,15]. Various experimental studies show that coating medical implants with these proteins leads to improvement in tissue integration and regeneration. These bioactive molecules, which functionalize PDMS in the form of a micro-pattern, have tunable impacts on the adhesion and the proliferation of cells on the substrates [16,17]. Recently, our study showed that genetically engineered filamentous bacteriophage can be introduced as an effective tool for biochemical surface modification, especially in targeted anti-inflammatory implant as well as in tissue engineering applications [18,19]. Such phages may act as linkers for the attachment of biomolecules to the implant materials to control the cellular responses. The fd-phage is a member of Ff filamentous phage family consisting of a circular single-stranded DNA (ssDNA) and a surrounding capsid protein. The capsid protein is mainly composed of the major coat protein, p8 (~2700–3000 copies), aligned along the ssDNA. Five copies of minor coat protein, p3, are located at the one end of the fd filament and five copies of p7 and p9 proteins are expressed at the other end [20]. H9c2 cardiomyocyte is currently used in vitro as a mimetic for skeletal and cardiac muscle due to its importance of highly organized cell orientation [21]. This topographical cue is major influence on skeletal muscle cell culture because the structure is long parallel bundles are formed by cell organization and differentiation [22]. In this regards, it is necessary to elucidate whether the use of cell patterning can be influenced on a cardiac morphology, cell alignments and the applications for future stent coating and patterning materials. Therefore, we aimed to fabricate more suitable environments that induce cell alignment and elongation by combining micro-scale patterns with organic coating materials. RGD-phage is an fd-tet derived phage of which RGD gene encoding recombinant p8 protein was inserted into the fd-tet plasmid. We hypothesized that the micro-patterning combined with the filamentous shaped and 3000 copies of RGD peptide displayed phage system may serve as a novel multi-functional platform and the cost-effective materials to study in vitro cell guidance and behaviors. In this study, we investigated the effect of the RGD-phage on the cellular response in the micro-patterns of PDMS. Cell adhesive molecules (RGD peptides) were genetically engineered on the P8 capsid on the phage for the improvement of cell binding and RGD-phage immobilization on 1D PDMS micro-patterns. We also compared the early response of H9c2 cardiomyocyte on as-deposited and RGD-phage coated PDMS micro-patterns for future stent coating materials.

## 2. Materials and Methods

### 2.1. Genetic Modification and Amplification of Phage

The recombinant fd-tet plasmid (kindly provided by Prof. Dr. Georg P Smith, University of Missouri, Columbia, MO, USA) and the N-terminus of recombinant p8 were used to introduce the genes encoding RGD for cell adhesion [19]. The sequences of adaptor DNA fragments were constructed using the complementary oligonucleotides as follows:

*RGD oligonucleotides*

Forward primer: 5′-AGCTTT TGT AGG GGT GAC GGT AGG TGC GGTAC-3′
Revers primer: 5′-C GCA CCA ACC GTC ACC CCT ACA AA-3′

Cell adhesive peptide (RGD) was introduced for the recombinant p8 of plasmid. The recombinant plasmid was digested by SfiI/NotI (New England Biolabs, Frankfurt am Main, Germany) to obtain the N-terminus of recombinant p8 genes. Briefly, each of the two complementary oligonucleotide mixtures was boiled for 10 min and cooled down to room temperature to produce an adaptor DNA. Isolation of wild type phages (wt-phage) and re-phage from the host cells was performed by the polyethylene glycol/sodium chloride (PEG/NaCl) precipitation method, as described previous study [18]. Afterwards growing the transformed K91BK cells were grown in Luria Broth supplemented with tetracycline (20 μg/mL) and kanamycin (100 μg/mL) at 37 °C with vigorous shaking (260 rpm) overnight. Final phage pellets were dialyzed with 30 kDa cut off membrane against 10 mM phosphate buffered saline solution (PBS) at pH 7.4 overnight to remove the remaining PEG. The phage concentration (colony-forming units per milliliter, cfu/mL) was determined by the phage titration. Phage suspensions were stored at 4 °C. The concentration was diluted with distilled-deionized water (ddH_2_O) to a phage stock concentration of 1 × 10^12^ cfu/mL. A photospectrometer (Evolution 60, UV/Vis) was used to estimate the concentration.

### 2.2. Micro-Patterned PDMS Substrates Fabrication

**Figure 1:**
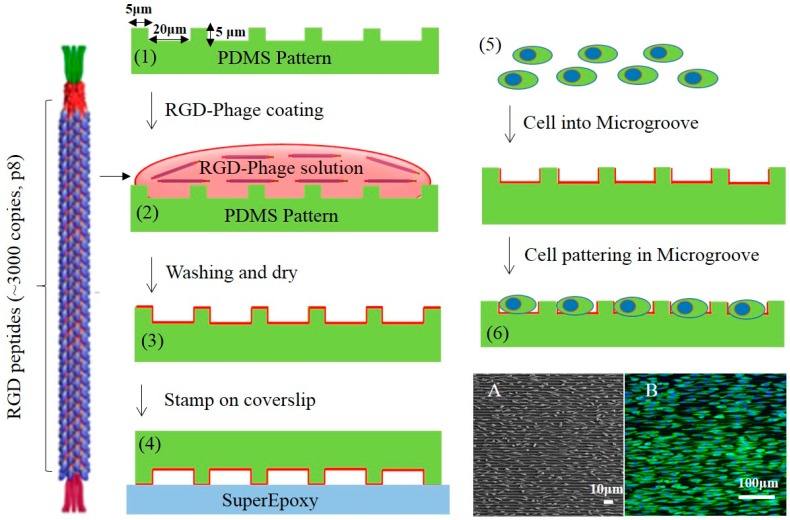
Schematic illustration of the fabrication method for RGD-Phage coating on 1D micro-patterned PDMS. Fluorescence and SEM (**A**) and Fluorescence images (**B**) of cell patterning in microgrooves.

Micro-patterned PDMS used to study cell adhesion and behavior was fabricated through standard photolithography. Briefly, a 4-inch silicon wafer was spin-coated with photoresist (SU-8 3010 resist) with thinner and then put into a soft bake oven for 2 min at 95 °C to remove the solvent. After the soft bake step, the wafer was exposed to ultraviolet light (MA6 Aligner2, UV Source 365 nm) under 100 mJ/cm^2^. The post-exposure bake was performed at 65 °C for 1 min and at 95 °C for 1 min. During development, the UV-exposed SU-8 layer was removed and the desired patterns were manufactured. The master substrate was coated with 10 µL of silane (Trichloro (1H,1H,2H,2H)-perfluorooctylsilane, Sigma-Aldrich, Saint Louis, MO, USA ) stored overnight under vacuum conditions to make detachment of PDMS from the template easier. To fabricate the PDMS molds containing microscale topographical patterns with 20 μm spacing, 5 μm width and 5 μm height of microgroove, polydimethylsiloxane (PDMS; Sylgard 184, Dow-Corning, Midland, MI, USA) was poured on the master surface, then cured in an oven at 70 °C for at least 2 h. The cured PDMS micro-pattern surfaces were cast with 1 × 10^12^ cfu/mL for 1 h to allow sufficient phage coating onto the underlying oxygen plasma treated PDMS surfaces. Subsequently, the phage-coated micro-pattern was stamped on super Epoxy glass to remove phage on the ridge of PDMS. Following an incubation of FITC conjugated anti-fd phage antibodies (1:5000, Sigma, Bandai, Fukushima, Japan) at 1 h at 37 °C, RGD-phages were observed by immunofluorescent staining. The micro-patterns were rinsed with PBS and maintained in a supplemented cell medium until cell seeding as shown in Figure 1. 

### 2.3. Cell Culture on Micro-Patterned Substrates

Rat H9c2 cardiomyocyte (ATCC: CRL-144, Rockville, NY, USA) was seeded on RGD-phage coated 1D micro-patterned substrates following the protocol as described previously [23]. Cardiomyocytes were incubated in culture using DMEM supplemented with 10% (*v*/*v*) fetal bovine serum (FBS), 100 U/mL penicillin G, 100 mg/mL streptomycin, and 2 mM l-glutamine at 37 °C with 5% CO_2_ supply. Flat PDMSs were used as a control. After 48 h of cell culture, the cells were fixed with 4% formaldehyde in PBS for 15 min. After being made permeable with 0.25% Triton X-100, samples were incubated with signal enhancer (Image-iTTM FX signal enhancer, Invitrogen, Carlsbad, CA, USA) to block the cells. Subsequently, the cells were treated for 1 h with mouse anti-vinculin (Sigma-Aldrich) and then washed three times with PBS. The cells were then treated with anti-mouse secondary antibody conjugated with Alexa 594 (Invitrogen Carlsbad, CA, USA,) for 1 h and then washed. For F-actin staining, the cells were treated with Alexa Fluor^®^ 488 Phalloidin (Invitrogen, Carlsbad, CA, USA) for 30 min. The samples were removed from the plate and placed on the slide glass with drops of mounting solution (mounting solution with DAPI, Vector). Finally, the staining was visualized with an Olympus IX51 inverted microscope with excitation wavelength at 550 and 660 nm, respectively. To evaluate cell proliferation, Cell counting kit-8 (CCK-8, Dojindo Laboratories, Kumamoto, Japan) and Image J software were used. 10 µL of CCK-8 solution was added to the 96-well plate containing cells seeded on PDMS surfaces. After 4 h of incubation, the supernatant was moved to another well plate to avoid the interference of PDMS in the measurement. The optical density was measured by using a microplate reader at 450 nm. The cell viability results are represented as the mean and standard deviation (±SD) from three independent experiments, with the significance of differences evaluated using ANOVA with Fisher's post hoc comparisons. *p* values of 0.05 were considered significant for all tests. 

### 2.4. Scanning Electron Microscope (SEM) Imaging

Substrates were washed 2 times with PBS at 37 °C and fixation was carried out using 2.5% glutardialdehyde in 0.5 M cacodylate buffer with 6% sucrose for 2 h at room temperature. After 10 min of incubation, the samples were washed with 0.1 M cacodylate buffer for 10 min 3 times. The samples went through post-fixation with 1% osmium tetroxide for 1 h and subsequently were dehydrated by a graded series of concentrations of ethanol (30%, 50%, 70%, 80%, 90%, 100% ×3 times) for 15 min each step. The dehydrated samples were placed in various concentrations of isoamyl acetate and then hexamethyldisilazane which was followed by dehydration. The substrates were subjected to critical point drying (Polaron CPD 7501, Quorom Technologies, Ringmer, UK). The samples were then sputtered with gold-palladium (Polaron, Sputter Coater, CA, USA) and visualized under SEM (Quanta FEI-SEM, Eindhoven Holland). 

### 2.5. Statistical Analysis

All data are expressed as ± standard error of mean (SEM). Data were analyzed statistically by ANOVA at a *p* < 0.05 confidence interval. 

## 3. Results and Discussion

### 3.1. Immobilization of RGD-Phage on PDMS Micro-Patterned Substrates

**Figure 2:**
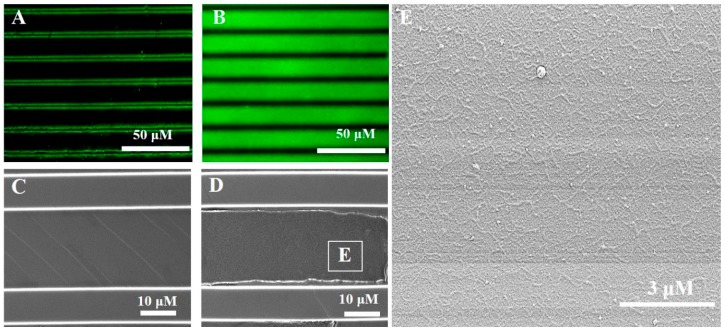
Fluorescence and SEM images of non-coated PDMS micro-patterns (**A**,**C**) and RGD-phage coated PDMS micro-grooves (**B**,**D**,**E**), respectively. Phages were stained with anti-M13 (green) antibody in 20 μm micro-grooves (**B**). Box in Figure 2D indicate film like re-phage assemblies (**E**).

As shown in Figure 2, fluorescence microscope and SEM analyses show the RGD-phage coated the groove of 1D PDMS micro-pattern. The topographical patterns with 20 μm spacing, 5 μm width and 5 μm height of microgroove was selected to optimize for cell fitting and immobilization. Through the high fluorescence intensity contrast it can be clearly seen that RGD-phages were successfully immobilized on PDMS grooves and were removed from the ridge after the micro-contact on Epoxy glass as shown in Figure 2B,D. In general, a protein has a higher affinity of the epoxy group activated glass than PDMS by multipoint covalent attachments [24]. FITC tagged anti-phage antibody was washed away from the non-coated PDMS pattern. There are dehydrated fluorescence were detected in both edges in the groove due to the reminder of non-specific antibody in washing solution (Figure 2A). In line with the observation, SEM image shows no phages in the groove of micro-pattern after washing step (Figure 2C) whereas, RGD-phage coated PDMS grooves result in self-assembled RGD-phage as shown in Figure 2D. We observed that the RGD-phages formed thin patch-like layers in the microgrooves as indicated by the inset in Figure 2D as shown in Figure 2E. This immobilization can be explained by a high-avidity binding of filamentous phage and the oxygen plasma treated PDMS from being a hydrophobic to a hydrophilic surface.

### 3.2. Cell Adhesion and Morphology Changes on Prepared Surfaces 

**Figure 3:**
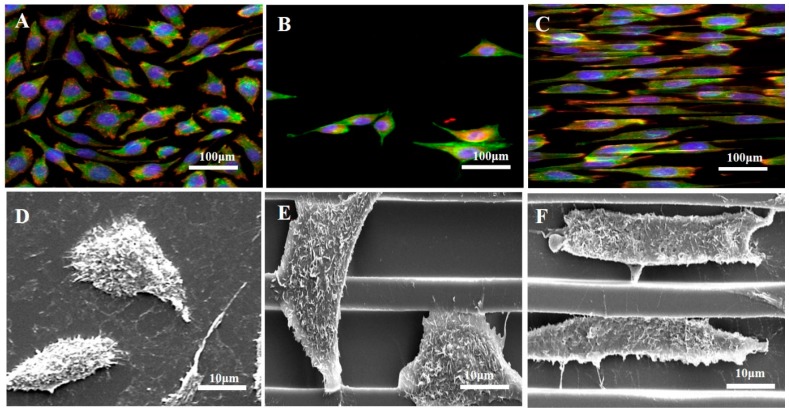
Fluorescence and SEM images of human endothelial H9c2 cardiomyocytes stained with F-actin (green), vinculin (red) and DAPI (blue) on flat PDMS (**A**,**D**), non-coated PDMS micro-patterns (**B**,**E**) and RGD-phage coated PDMS micro-patterns (**C**,**F**) after 72 h, respectively.

In order to validate the effect of the phage coated microgroove on cell behavior, the cell morphological changes and adhesion were investigated through the observation of the focal adhesion on the non-coated and RGD-phage coated 1D PDMS micro-pattern as shown in Figure 3. Cell adhesion markers, F-actin and Vinculin, indicated that cells are strongly attached with RGD-phage coated PDMS microgrooves with cell spreading. Fluorescent microscope images show that the cells were placed on the microgroove with alignment to the phage coated PDMS pattern (Figure 3C). As expected, the cells on the non-patterned reference and the non-coated PDMS pattern spread over the surface with a random orientation (Figure 3A,B). Meanwhile, phage coated PDMS patterns showed higher orientation with elongated morphology after 72 h cell culture. In addition, scanning electron microscopy (SEM) images revealed that the cells aligned along the PDMS strip and were located in the microgroove (Figure 3F), indicating that the phage coated micro-pattern has a greater effect on the extent of cell alignment as well as the morphology of cells in comparison to the non-coated micro-pattern (Figure 3E). 

### 3.3. Cell Proliferation on Micro-Grooved Surfaces

**Figure 4:**
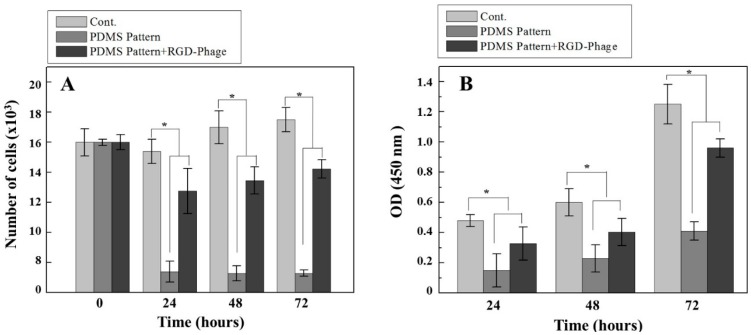
Cell proliferation rate as determined by cell counts (**A**) and CCK-8 assay (**B**). Cell number was determined using Image J (The number of cells per unit cm^2^). Data were analyzed statistically by ANOVA at a *p* < 0.05 confidence interval and Fisher’s LSD test. Asterisk denotes differences of the populations (* *p* < 0.05 compared to respective controls).

Cell proliferation and viability results from 0 to 72 h cell cultures are shown in Figure 4. After the cell alignment after static seeding for 0, 24, 48 and 72 h, the cells were quantified using Image J software to determine the number of cells as depicted in Figure 4A. The cell density, the number of cells per unit cm^2^, was calculated. The initial cell cultures (0 h) showed a similar number of cells (15 × 10^3^ cells/cm^2^). Compared to flat PDMS at a cell density around 15 × 10^3^ cells/cm^2^ as control, the cell density was observed around 2.0 × 10^3^ cells/cm^2^ in non-coated PDMS micro-patterns and the cell density around 10 × 10^3^ cells/cm^2^ were observed in RGD-phage coated PDMS micro-patterns after 24 h cell culture which is quite similar to 48 and 72 h. In contrast to significant decreases of cell density in non-coated PDMS micro-pattern, relatively higher cell density was observed in RGD-phage coated PDMS micro-patterns. Likely, compared to cell viability of non-coated micro-patterns, RGD-phage coated micro-patterns led to a clear increase in cell viabilities as shown Figure 4B. We observed a decrease in cell viability and population on RGD-phage coated micro-patterns even in comparison to cells cultured on flat PDMS as a control. This decreases in cell density and viability may be explained by geometrically constrained barriers, as these individually separated microgrooves cannot provide enough contact for cell to surface interactions. On the other hand, the cell growth within the micro-groove gave a clear direction to the RGD-phage induced cell organization. Our results suggest that there is a direct correlation between cell attachment and RGD-displaying phage in micro-grooves. Through our observation, we demonstrated that RGD-displaying phage provided an environment that can support H9c2 cardiomyocyte to efficiently organize cellular morphogenesis as well as additional micro-patterned topographical cues to control the cell length and size which can induce the bundles like architecture for the simulation of multi-strip cardiac muscle. 

## 4. Conclusions

We successfully demonstrated incorporating genetically engineered phage assemblies on 1D micro-patterned groove. The 1D micro-patterned substrates functionalized with RGD-phage influenced both the initial location and orientation of cells. It provided an enhanced H9c2 cardiomyocyte adhesion and alignment, which can be interesting for future stent coating materials in cardiology. More importantly, our approach using recombinant RGD-displayed phages can be genetically engineered to produce both metallic binding motif as well as biological function on future metal alloy stent applications.
